# The five primary prostaglandins stimulate contractions and phasic activity of the urinary bladder urothelium, lamina propria and detrusor

**DOI:** 10.1186/s12894-020-00619-0

**Published:** 2020-04-29

**Authors:** Zane Stromberga, Russ Chess-Williams, Christian Moro

**Affiliations:** Centre for Urology Research, Faculty of Health Sciences and Medicine, Bond University, Gold Coast, Queensland 4226 Australia

**Keywords:** Inflammation, Prostaglandins, Urinary bladder, Urothelium, Detrusor

## Abstract

**Background:**

Inflammation is often associated with several bladder dysfunctions, including overactive bladder (OAB) and interstitial cystitis/bladder pain syndrome (IC/PBS). As such, inflammation of the bladder and the actions of inflammatory mediators may contribute to the development of urinary symptoms. This study assessed the actions of PGE_2_, PGF_2_, PGD_2_, TXA_2_, and PGI_2_ on urinary bladder urothelium with lamina propria (U&LP), and detrusor smooth muscle.

**Methods:**

Studies were carried out using isolated tissue baths, where strips of porcine bladder U&LP or detrusor were exposed to varying concentrations of prostaglandin agonists (1 μM and 10 μM).

**Results:**

All assessed prostaglandin agonists contracted both the U&LP and detrusor smooth muscle, with the rank order of contractile response effectiveness as: PGE_2_ > PGF_2α_ > TXA_2_ > PGD_2_ > PGI_2_. In U&LP, treatment with PGE_2_ (10 μM) increased tonic contractions by 1.36 ± 0.09 g (*n* = 42, *p* < 0.001) and phasic contractions by 40.4 ± 9.6% (*n* = 42, *p* < 0.001). In response to PGF_2α_ (10 μM), U&LP tonic contractions increased by 0.79 ± 0.06 g (*n* = 14, *p* < 0.001) and phasic activity by 13.3% ± 5.3% (*n* = 15, *p* < 0.05). In detrusor preparations, PGE_2_ (10 μM) increased tonic contractions by 1.32 ± 0.13 g (*n* = 38, *p* < 0.001) and PGF_2α_ (10 μM) by 0.97 ± 0.14 g (*n* = 12, *p* < 0.001). Only 34% (*n* = 48) of all detrusor preparations exhibited spontaneous activity prior to the addition of any agonist at a frequency of 2.03 ± 0.12 cpm. In preparations that did not exhibit initial phasic activity, all of the prostaglandin agonists were capable of commencing phasic activity.

**Conclusions:**

The urinary bladder U&LP and detrusor respond to a variety of prostaglandin agonists, with their activation resulting in direct contractions, as well as increases to spontaneous contractile activity. This study presents the prostaglandin receptor system as a potential therapeutic target for lower urinary tract dysfunction.

## Background

The involvement of prostaglandins in bladder physiology was first recognised from their release after urinary bladder distension or injury to the urothelium [1, 2]. An increase of prostaglandins in the urine of patients suffering from OAB has been well-reported previously [3–6], suggesting the prostaglandin system as a potential future therapeutic target in various bladder dysfunctions. The exact role and mechanisms of endogenous prostaglandins in the urinary bladder are not well understood. However, previous studies utilising exogenous prostaglandins have shown that these chemicals can alter contractility and micturition reflex in human bladders [7].

Prostaglandin production is generally low in healthy tissue but can increase immediately following acute inflammation [8]. They are synthesised in the bladder by cyclooxygenase (COX) and then subsequently converted into five primary prostanoids via their respective synthases: PGE_2_, PGD_2_, PGF_2α_, prostacyclin (PGI_2_) and thromboxane (TXA_2_) [9]. Prostaglandins are synthesised in both the bladder urothelium with lamina propria (U&LP) and in detrusor smooth muscle in response to stretch, nerve stimulation, U&LP damage or other inflammatory mediators [10, 11]. The production of prostaglandins is determined by the cells present at sites of inflammation capable of synthesising prostaglandins and the activity of the two cyclooxygenase isoenzymes, namely COX-1 and COX-2. For example, macrophages predominantly generate PGE_2_ and TXA_2_, whereas mast cells produce PGD_2_ [12]. COX-1 is present in most cells, whereas the expression of COX-2 is generally low in cells, but can increase dramatically upon stimulation by immune cells [13]. Prostaglandin I_2_ is the main prostaglandin synthesised in the human bladder, followed by PGE_2,_ PGF_2α_ and TXA_2_ [14, 15].

Although past studies have explored the effects of the different prostaglandins on the urinary bladder with a large focus on the actions of PGE_2_, a complete understanding of the contractile effects of the other four prostaglandins on the urinary bladder remain unclear. Specifically, of interest is to determine how the actions of the prostaglandins affect urothelium with lamina propria that is separated from the detrusor smooth muscle. Therefore, this study aimed to assess the influence of PGE_2_, PGF_2α_, PGD_2_, TXA_2_ and PGI_2_ on the urinary bladder urothelium with lamina propria and detrusor smooth muscle contractions and phasic activity.

## Methods

### Tissue preparation

Urinary bladders were obtained from Large White-Landrace pigs (approximately six months old, weighing between 80 and 100 kg) from the local abattoir after slaughter for the routine commercial provision of food. All methods were carried out in accordance with relevant Australian guidelines and regulations, and all experimental protocols were in accordance the Australian Code of Practice for the Care and Use of Animals for Scientific Purpose [16]. As no animals were bred, harmed, culled, interfered, or interacted with as part of this research project, Animal Ethics Approval was not required for offal use [17]. Urothelium with lamina propria was dissected from the underlying detrusor layer, consistent with methods carried out in past studies [18–21], and cut in strips. Adjacent strips of U&LP and detrusor (10 mm × 5 mm) were tied vertically between an isometric force transducer (MCT050/D, ADInstruments, Castle Hill, Australia) and a fixed hook in 10 mL organ baths (Labglass, Brisbane, Australia), and superfused with Krebs-bicarbonate solution (NaCl 118.4 mM, NaHCO_3_ 24.9 mM, CaCl_2_ 1.9 mM, MgSO_4_ 2.41 mM, KCl 4.6 mM, KH_2_PO_4_ 1.18 mM and D-glucose 11.7 mM) and carbogen gas (95% oxygen and 5% carbon dioxide) at 37 °C. After tissue mounting, strips of U&LP and detrusor were washed three times, tension adjusted to 1.5–2.0 g and tissues left to equilibrate for 30 min. After the equilibration period, a single dose of a prostaglandin receptor agonist was added to the tissue strip.

### Pharmaceutical agents

The following compounds were used in this study: prostaglandin E_2_, prostaglandin F_2α_, prostaglandin D_2_, prostaglandin I_2_ and thromboxane A_2_ (U-46619, Cayman Chemicals, Michigan, USA). Prostaglandin E_2_, prostaglandin F_2α_, prostaglandin D_2,_ and prostaglandin I_2_ were dissolved in 100% ethanol and diluted with distilled H_2_O. U-46619 was supplied as a solution in methyl acetate, which was diluted with distilled H_2_O. Two concentrations of each prostaglandin receptor agonists were selected, 1 μM and 10 μM.

### Data analysis

Data were graphed and analysed using GraphPad Prism version 8.3 for Windows (GraphPad Software, La Jolla, California, USA). Statistical analysis was conducted using a paired Student’s *t*-test, where *p* < 0.05 was considered as significant. All values were reported as mean change ± SEM. *n* equates to the number of individual bladders used in this study.

## Results

### Prostaglandin agonists for increasing U&LP spontaneous phasic activity

**Fig. 1:**
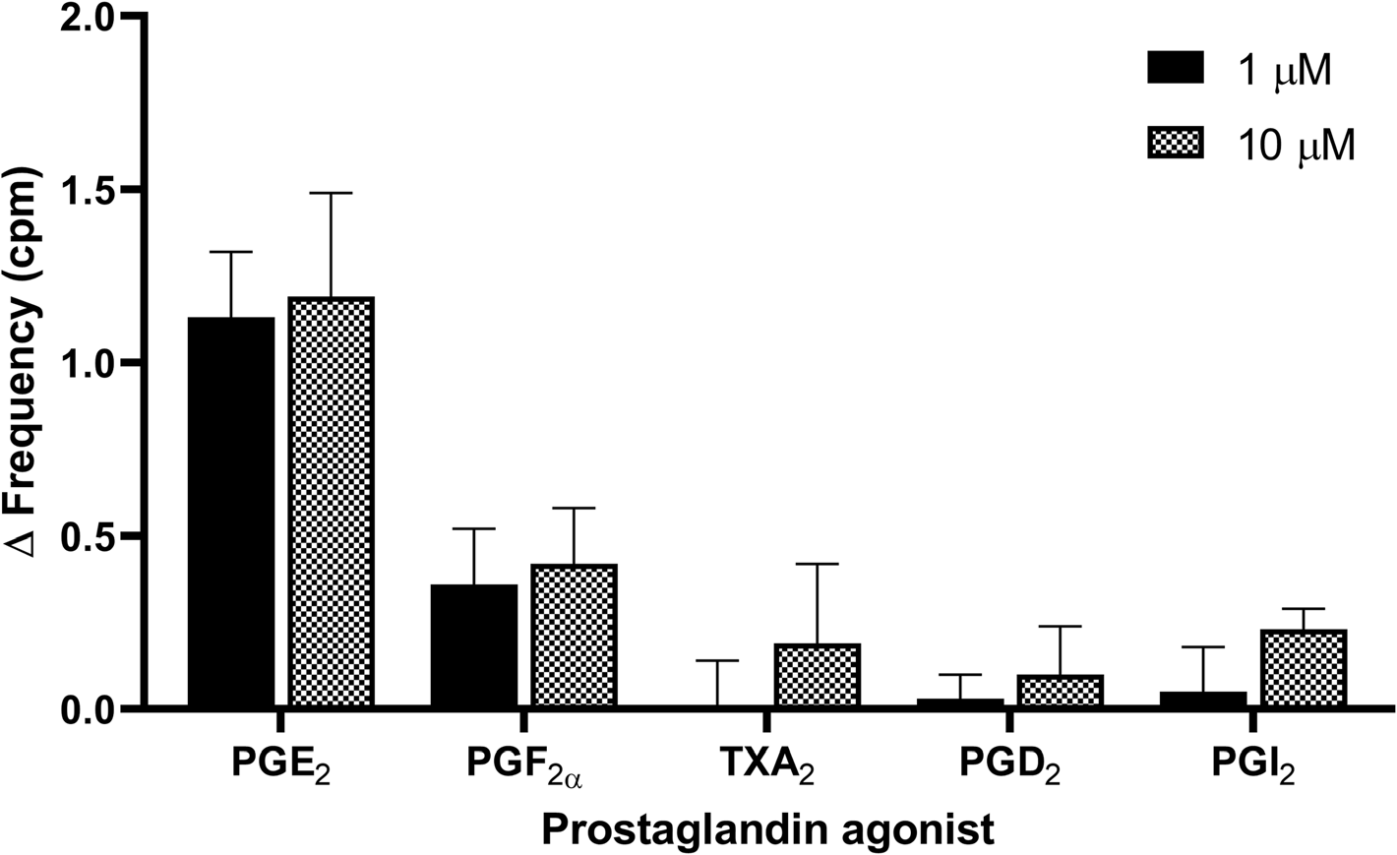
U&LP changes in the frequency of spontaneous phasic contractions after the treatment with 1 μM and 10 μM of each specific prostaglandin agonists E_2_, F_2α_, TXA_2_, D_2_, and I_2_. There were no statistically significant differences in frequency changes between the 1 μM and 10 μM concentrations for any of the agonists (unpaired Student’s 2-tailed *t*-test)

Strips of U&LP exhibited spontaneous phasic contractions in the absence of any stimulation at a mean frequency of 3.26 ± 0.07 cycles per minute (cpm, *n* = 146). Treatment with PGE_2_ caused the most prominent increases to U&LP spontaneous contractile activity. When PGE_2_ (1 μM) was added to isolated tissues, spontaneous activity increased by 39.2% ± 6.7% (*n* = 38, *p* < 0.001, Fig. 1). A greater concentration of PGE_2_ (10 μM) showed similar increases of 40.4% ± 9.6% to the U&LP spontaneous activity (*n* = 42, *p* < 0.001). Treatment with PGF_2α_ showed smaller increases of 10.5% ± 4.6% to spontaneous activity when treated with 1 μM (*n* = 10, *p* < 0.05) and 13.3% ± 5.3% when treated with 10 μM (*n* = 14, *p* < 0.05). The addition of PGI_2_ (10 μM) increased spontaneous activity by 6.2% ± 1.6% (*n* = 8, *p* < 0.01) but had no effect at a lower concentration (1 μM, *n* = 8). The frequency was not significantly affected by PGD_2_ (1–10 μM, *n* = 12) or TXA_2_ (1–10 μM, *n* = 16).

The average amplitude of these spontaneous phasic contractions exhibited in U&LP strips in the absence of any stimulation was 0.57 ± 0.02 g (*n* = 146). In response to treatment with 1 μM PGE_2_, amplitude decrease of 0.14 ± 0.04 g (*n* = 38, *p* < 0.001, Table 1) were observed. Similar decreases of 0.16 ± 0.03 g were also observed in response to a higher PGE_2_ concentration (10 μM, *n* = 42, *p* < 0.01). Treatment with TXA_2_ (1 μM) showed a significant decrease in the amplitude by 0.28 ± 0.06 g (*n* = 8, *p* < 0.01), which was not observed at a higher concentration (10 μM, *n* = 6). The addition of PGI_2_ (10 μM) decreased amplitude of spontaneous activity by 0.14 ± 0.05 (*n* = 8, *p* < 0.05) but had no effect at a lower concentration (1 μM, *n* = 8). The amplitude of spontaneous contractions was not altered by the addition of either PGF_2α_ (1–10 μM, *n* = 24) or PGD_2_ (1–10 μM, *n* = 12, Table 1). None of the decreases in the amplitude of spontaneous phasic contractions of the U&LP were significantly affected by the two different prostaglandin receptor agonist concentrations (1 μM and 10 μM).

**Table 1:** U&LP changes in the amplitude of phasic contractions in response to the five primary prostaglandin agonists (mean ± SEM)

	1 μM of agonist			10 μM of agonist		
Agonist	Absence (g)	Presence (g)	n	Absence (g)	Presence (g)	n
PGE_2_	0.53 ± 0.05	0.40 ± 0.03***	38	0.53 ± 0.04	0.37 ± 0.03**	42
PGF_2α_	0.30 ± 0.03	0.29 ± 0.01	10	0.51 ± 0.06	0.46 ± 0.08	14
TXA_2_	0.90 ± 0.16	0.62 ± 0.14**	8	0.75 ± 0.16	0.71 ± 0.25	6
PGD_2_	0.59 ± 0.10	0.46 ± 0.04	4	0.55 ± 0.08	0.43 ± 0.06	8
PGI_2_	0.64 ± 0.07	0.56 ± 0.07	8	0.57 ± 0.09	0.43 ± 0.06*	8

*p < 0.05, **p < 0.01, ***p < 0.001. Paired Student’s *t*-test

### Prostaglandin agonists in stimulating phasic contractions in detrusor

Total of 34% (*n* = 48) of the detrusor preparations that were set up in the organ baths exhibited spontaneous activity prior to the addition of any agonists. These contractions occurred at an average frequency of 2.03 ± 0.12 cpm (*n* = 48) with an average amplitude of 0.26 ± 0.02 g (*n* = 48). However, the majority of the detrusor preparations, that were otherwise quiescent developed spontaneous phasic contractions after the addition of the agonist.

Of those detrusor preparations that did not exhibit initial phasic activity during baseline: PGE_2_ (1 μM) sparked contractions in 68% of preparations (*n* = 19) and PGE_2_ (10 μM) in 69% (*n* = 22); PGF_2α_ (1 μM) initiated contractions in in 56% (*n* = 5) and PGF_2α_ (10 μM) in 88% (*n* = 7); TXA_2_ (1 μM) initiated contractions in 63% (*n* = 5) and TXA_2_ (10 μM) in 80% (*n* = 4); PGD_2_ (1 μM) initiated phasic activity in 50% (*n* = 2) and PGD_2_ (10 μM) in 75% (*n* = 6); and lastly PGI_2_ (10 μM) initiated contractions in 40% (*n* = 2) of preparations. This demonstrates the ability of prostaglandin agonists to induce spontaneous activity in otherwise quiescent detrusor tissue strips.

### Prostaglandin agonists in stimulating tonic contractions in U&LP

**Fig. 2:**
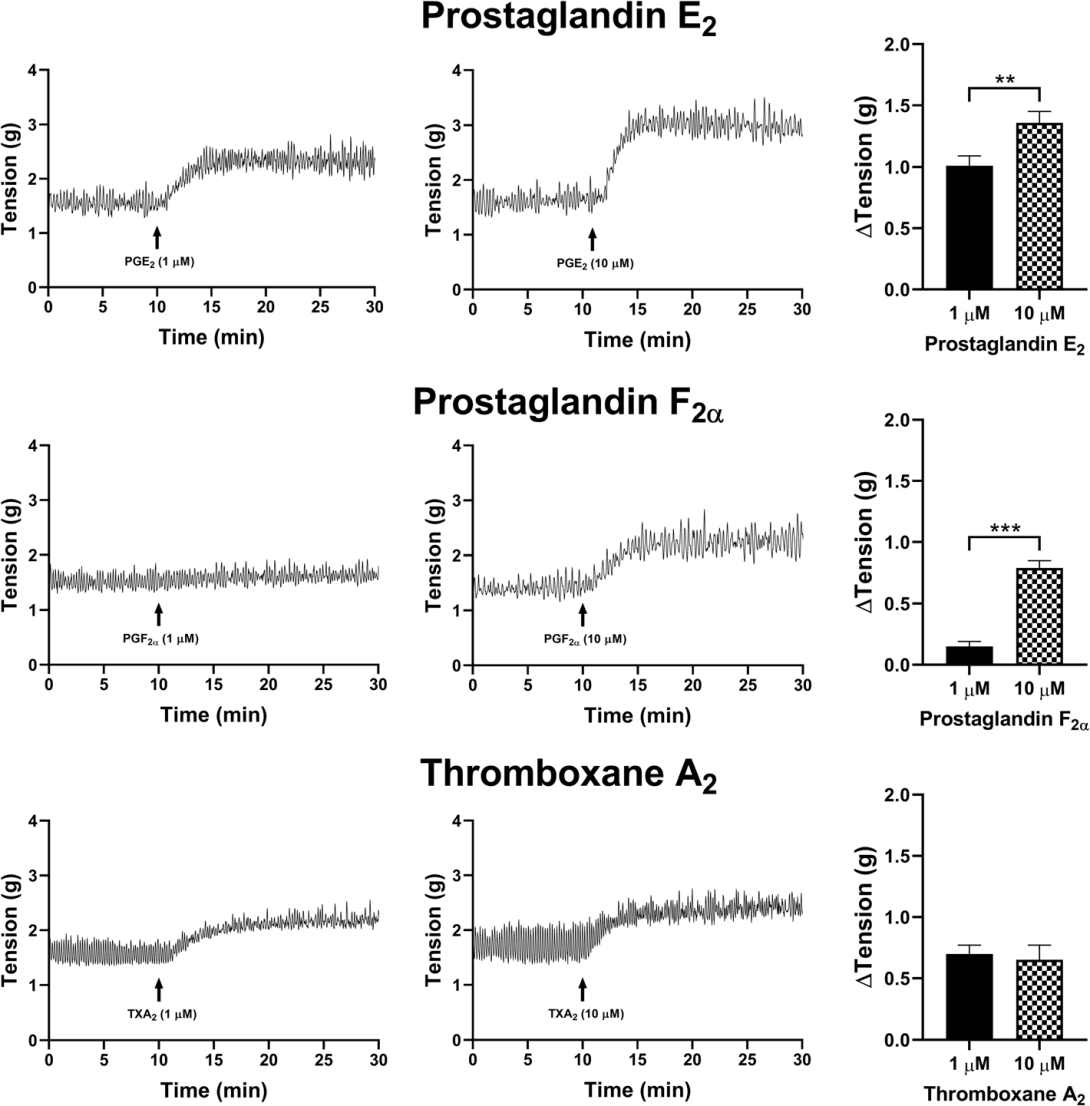
U&LP changes in tonic contractions in response to the treatment with 1 μM and 10 μM of prostaglandin E_2_ (*top row*), F_2α_ (*middle row*) and TXA_2_ (*bottom row*). Sample traces of the responses observed to two concentrations of prostaglandin agonist (*left & middle columns*). Increases in tonic contractions after treatment with each agonist are represented as mean change ± SEM (*right column*). Significant changes in the tonic contractions between 1 μM and 10 μM were evaluated using an unpaired Student’s two-tailed *t*-test, where *p < 0.05, **p < 0.01, ***p < 0.001

All assessed prostaglandin agonists contracted the U&LP with the rank order of contractile response effectiveness as: PGE_2_ > PGF_2α_ > TXA_2_ > PGD_2_ > PGI_2_. The addition of PGE_2_ (1 μM) to isolated U&LP induced tissue contractions, with increases of 1.01 ± 0.08 g (*n* = 38, *p* < 0.001) to the tonic contractions. When a greater concentration of PGE_2_ (10 μM) was selected, increases of 1.36 ± 0.09 g (*n* = 42, *p* < 0.001, Fig. 2) were observed. Treatment with 1 μM PGF_2α_ showed a small increase to tonic contractions of 0.15 ± 0.04 g (*n* = 10, *p* < 0.01) when compared to a higher concentration of 10 μM, which exhibited increases of 0.79 ± 0.06 g (*n* = 14, *p* < 0.001). The addition of two concentrations of TXA_2_ induced similar contractions, where tonic contraction increased by 0.70 ± 0.07 g when treated with 1 μM (*n* = 8, *p* < 0.001), and by 0.65 ± 0.12 g after treatment with 10 μM (*n* = 6, *p* < 0.001).

**Fig. 3:**
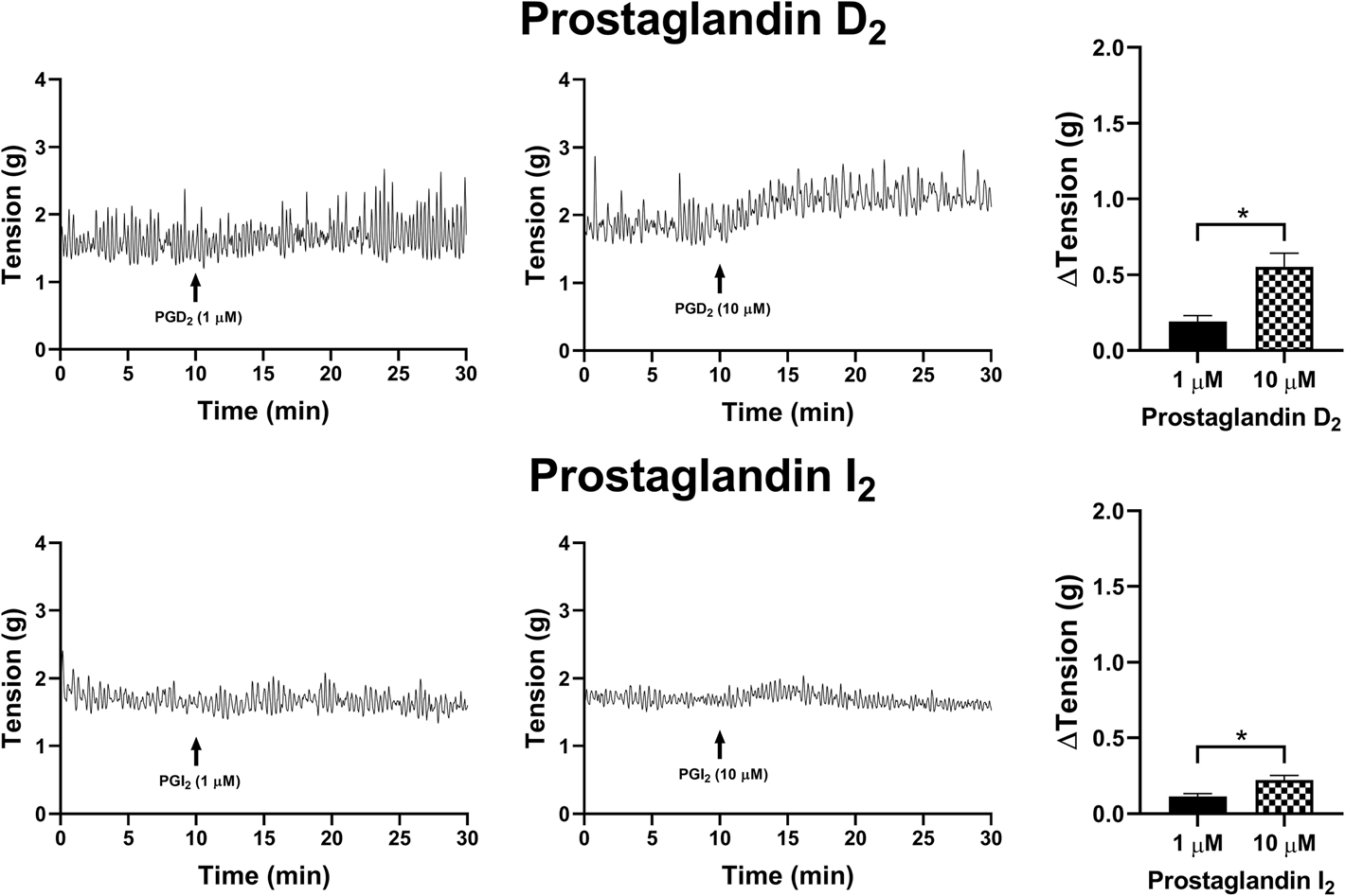
U&LP changes in tonic contractions in response to the treatment with 1 μM and 10 μM of prostaglandin agonists D_2_ (*top row*) and I_2_ (*bottom row*). Sample traces of the responses observed to two concentrations of prostaglandin agonist (*left & middle columns*). Increases in tonic contractions after treatment with each agonist are represented as mean change ± SEM (*right column*). Significant changes in the tonic contractions between 1 μM and 10 μM were evaluated using an unpaired Student’s two-tailed *t*-test, where *p < 0.05, **p < 0.01, ***p < 0.001

When PGD_2_ (1 μM) was added to the U&LP tissue preparations, tonic contractions increased by 0.19 ± 0.04 g (*n* = 4, *p* < 0.05, Fig. 3). Treatment with a higher concentration of PGD_2_ (10 μM) exhibited increases of 0.63 ± 0.09 g (*n* = 8, *p* < 0.001). The addition of PGI_2_ showed small increases in tonic contractions of 0.11 ± 0.02 g in response to 1 μM PGI_2_ (*n* = 8, *p* < 0.001), and 0.22 ± 0.03 g in response to 10 μM PGI_2_ (*n* = 8, *p* < 0.001, Fig. 3).

### Prostaglandin agonists in stimulating tonic contractions in detrusor

**Fig. 4:**
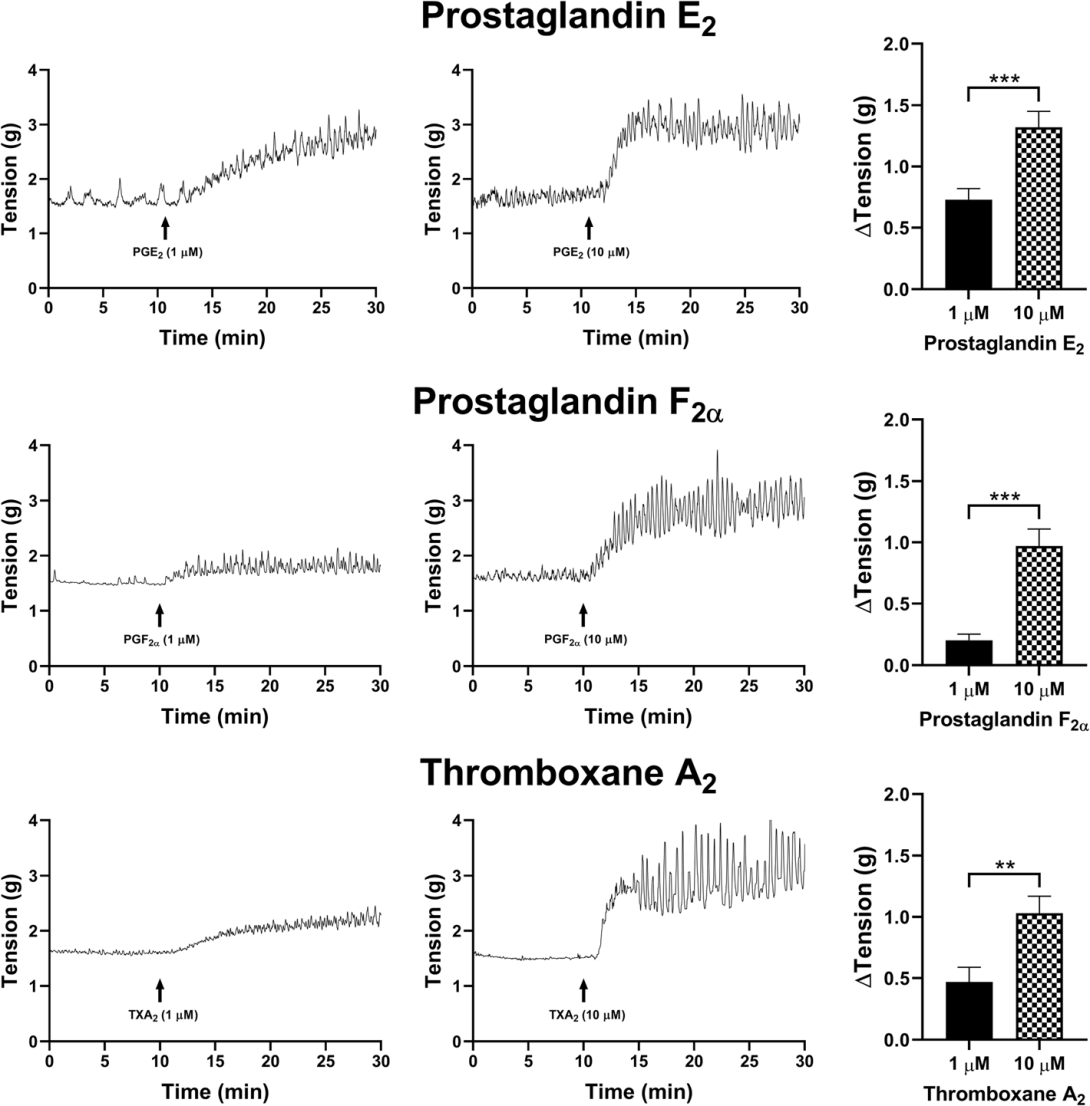
Detrusor changes in tonic contractions in response to the treatment with 1 μM and 10 μM of prostaglandin E_2_ (*top row*), F_2α_ (*middle row*) and TXA_2_ (*bottom row*). Sample traces of the responses observed to two concentrations of prostaglandin agonist (*left & middle columns*). Increases in tonic contractions after treatment with each agonist are represented as mean change ± SEM (*right column*). Significant changes in the tonic contractions between 1 μM and 10 μM were evaluated using an unpaired Student’s two-tailed *t*-test, where *p < 0.05, **p < 0.01, ***p < 0.001

All assessed prostaglandin agonists contracted the detrusor smooth muscle preparations with the rank order of contractile response effectiveness as: PGE_2_ > PGF_2α_ > TXA_2_ > PGD_2_ > PGI_2_. In detrusor preparations, PGE_2_ (1 μM) increased the tonic contractions by 0.73 ± 0.09 g (*n* = 34, *p* < 0.001), whereas PGE_2_ (10 μM) nearly doubled the response, producing an average increase of 1.32 ± 0.13 g (*n* = 38, *p* < 0.001, Fig. 4). Treatment with 1 μM PGF_2α_ showed a small increase of 0.20 ± 0.05 g (*n* = 10, *p* < 0.01), whereas 10 μM of PGF_2α_ increased the tonic contractions by 0.97 ± 0.14 g (*n* = 12, *p* < 0.001). When TXA_2_ was added, tonic contractions increased by 0.47 ± 0.12 g when treated with 1 μM (*n* = 8, *p* < 0.001), and by 1.03 ± 0.14 g (*n* = 6, p < 0.001, Fig. 4) when treated with 1 μM TXA_2_.

**Fig. 5:**
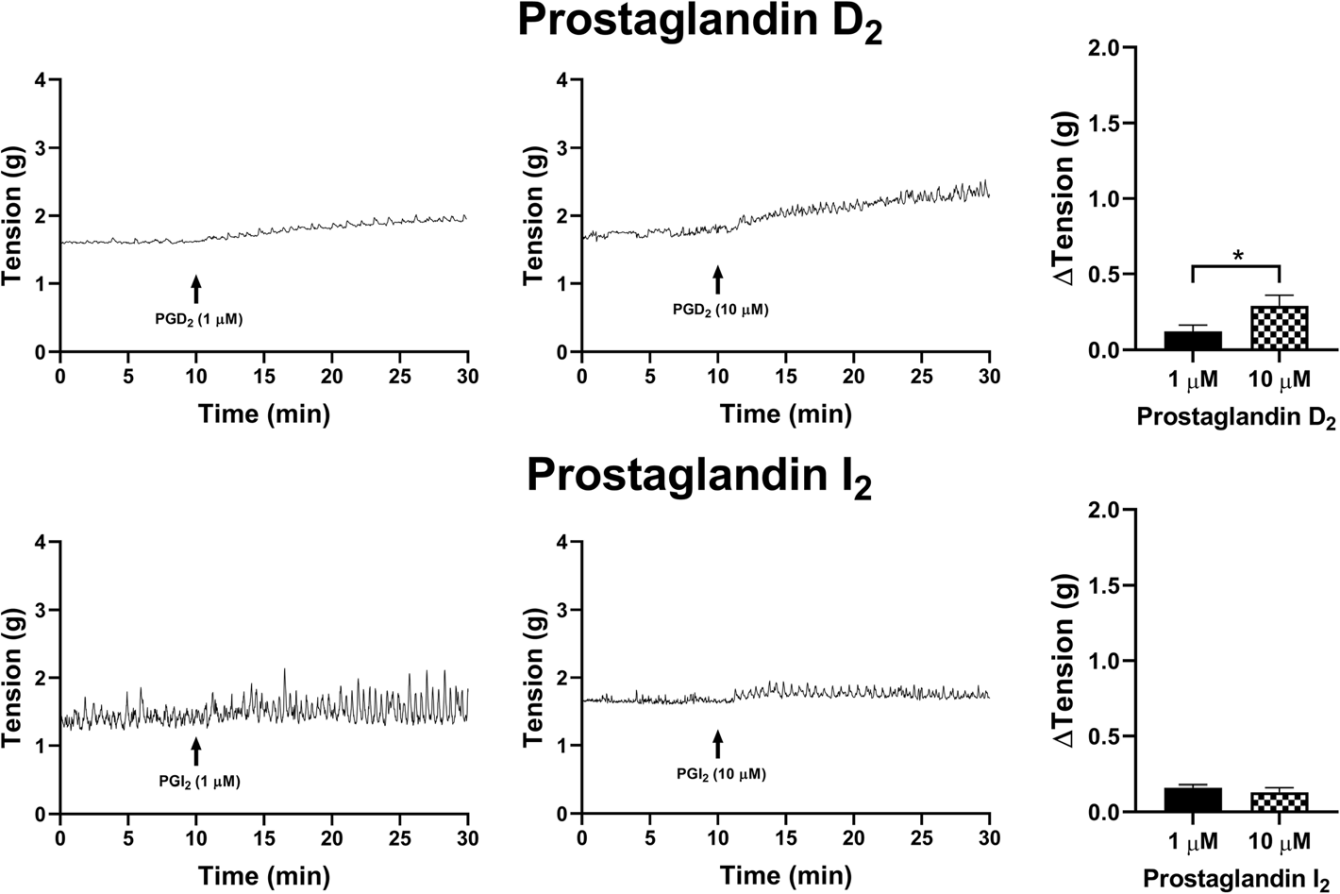
Detrusor changes in tonic contractions in response to the treatment with 1 μM and 10 μM of prostaglandin agonists D_2_ (*top row*) and I_2_ (*bottom row*). Sample traces of responses observed to two concentrations of a prostaglandin agonist (*left & middle columns*). Increases in the tonic contractions after treatment with an agonist are represented as mean change ± SEM (*right column*). Significant changes in the tonic contractions between 1 μM and 10 μM were evaluated using an unpaired Student’s *t*-test, where *p < 0.05, ***p < 0.001

PGD_2_ showed a small increase in the tonic contractions of 0.12 ± 0.04 g when 1 μM was added (*n* = 4, *p* < 0.05), and an increase of 0.36 ± 0.06 g when 10 μM PGD_2_ was added (*n* = 6, p < 0.01, Fig. 5). PGI_2_ showed small increases in tonic contractions at both concentrations, showing an increase of 0.16 ± 0.02 g when treated with 1 μM (*n* = 8, *p* < 0.001), and 0.13 ± 0.03 g when treated with 10 μM PGI_2_ (*n* = 8, *p* < 0.001, Fig. 5). The effects of prostaglandin agonists on tonic contractions of the detrusor smooth muscle were significantly different between the two concentrations (1 μM and 10 μM) for PGE_2_ (*p* < 0.001), PGF_2α_ (*p* < 0.001) and PGD_2_ (*p* < 0.05).

## Discussion

Previous research has shown that stimulation of the M3 muscarinic receptor in U&LP causes immediate contractions, as well as increases in the frequency of spontaneous phasic contractions, and reduction in their amplitude [22, 23]. In our study, the prostaglandin agonists have shown similar contractile responses to both tonic contractions and spontaneous activity, thereby associating the actions of prostaglandins with many of the bladder contractile dysfunctions, such as OAB and IC/BPS.

The ability to contract the tissue was varied between the different prostaglandin agonists. The rank order of agonist response in stimulating contractions in U&LP and detrusor was: PGE_2_ > PGF_2α_ > TXA_2_ > PGD_2_ > PGI_2_. This furthers previous research which reported the involvement of PGE_2_ in the initiation of micturition in both humans and animals [24], suggesting a contribution to bladder overactivity. Treatment with PGF_2α_ showed minimal increases at a concentration of 1 μM, yet responses were significantly enhanced in both U&LP and detrusor when increased to 10 μM. At the smaller concentration of 1 μM, treatment with TXA_2_ reached maximal contractile responses, and as such, was not enhanced at the higher agonist concentration of 10 μM. This was not the case with detrusor preparations, wherein the higher concentration of TXA_2_ (10 μM) resulted in significantly enhanced contractions. The responses observed in porcine tissue in response to PGF_2α_, and TXA_2_ are consistent with the Palea [25] findings. In addition, our study has established that U&LP isolated tissue is also capable of responding and producing definite increases in tonic contractions in response to these prostaglandin agonists.

Of the five prostaglandins, PGD_2_ and PGI_2_ had the smallest effect on both tonic contractions and spontaneous activity. This lack of increases to the tonic contractions or spontaneous contractile frequency may be explained by PGD_2_ having potential inhibitory actions via the stimulation of DP receptor [26]. An explanation for the small contractile effects observed in our study in response to PGI_2_, the main prostaglandin synthesised in the human bladder [14, 27], is that the aqueous solutions of PGI_2_ are extremely chemically unstable with a relatively short half-life, depending on the buffer concentration [28]. As such, future studies utilising more chemically stable PGI_2_ agonist analogous might provide further insights into the actions of this inflammatory mediator on the urinary bladder.

## Conclusions

The urinary bladder is capable of responding to all five major prostaglandins produced in the urinary bladder. Out of these prostaglandins, PGE_2_ and PGF_2α_ had the most significant impact on both contraction and increases to the spontaneous contractile frequency in the U&LP. All five prostaglandin receptor agonists were also capable of inducing spontaneous phasic contractions in otherwise quiescent detrusor tissue strips.

## Data Availability

The datasets used and/or analysed during the current study are available from the corresponding author on reasonable request.

## Abbreviations

PG: Prostaglandin
IC/BPS: Interstitial cystitis/bladder pain syndrome
OAB: Overactive bladder
U&LP: Urothelium and lamina propria
TX: Thromboxane
